# Supplementary material for: The propagation of admixture-derived adaptive radiation potential
Source: Proc Biol Sci. 2020 Sep 9;287(1934):20200941. doi: 10.1098/rspb.2020.0941 (PMC7542789; doi:10.1098/rspb.2020.0941)
Supplement: Supplementary Appendix [file rspb20200941supp1.pdf]

Supplementary Information

“The propagation of admixture-derived adaptive radiation potential”

Kagawa, K. & Seehausen, O.

Appendix S1. *The model*

We constructed an individual-based computer simulation model based on the model proposed in Kagawa & Takimoto (2018). The model simulates ecological and evolutionary dynamics of diploid organism with non-overlapping generations. Individuals are either female or male. The model considers evolution of a single quantitative ecological trait value x, which is controlled by L loci with additive effects in a genome consisted of $2n$ chromosomes (we used $n = 15$). Each locus is 5000 base-pairs (bps) long and each chromosome is l bps long. Locations of L loci along the genome were randomly assigned prior to each simulation run as follows: the genome, which is nl bps long in total, were divided into short segments of length 5000 bps and then L loci were randomly selected from all segments. Trait value of an individual i is given by $x_i = \sum_{k=1}^{L}(e_{ki1} + e_{ki2})$, where e_{ki1} and e_{ki2} are phenotypic effect values of two alleles of the locus k. Point mutations, which occur at rate μ per locus in meiosis, alter phenotypic effect values of alleles. The total number of mutations occurring at a meiosis is drawn from a Poisson distribution Po (μL) and then, target locus of each mutation is randomly assigned. Every derived nucleotide has a unique randomly assigned position within its target locus. Each allele carries a set S of derived nucleotides and a mutation on the allele adds a new derived nucleotide to S. Phenotypic effect of each derived nucleotide follows the normal distribution N (0, σ_m^2). Phenotypic effect value of an allele, e_{kij}, is given by $\sum_{u \in S_{kij}} \varepsilon_u$ where S_{kij} is the set of derived nucleotides carried by the allele and ε_u is the effect of the derived nucleotide u. In addition to mutations, crossover recombination, which occurs at a rate r per bp in meiosis, creates chromosomes with novel combinations of derived nucleotides. The total number of crossovers occurring in a meiosis is drawn from Po (rnl) and then location of each crossover point along the genome is assigned randomly. In the simulation implementation, we approximated positions along the genome given in base-pair units with double-precision floating-point numbers; positions in each chromosome ranging from 0 to l (in our simulations, $l = 2\times10^8$) base-pairs were mapped to double-precision floating-point numbers ranging from 0 to 2×10^2. Thus, our simulation implementation is based on the infinite sites model.

The model considers multiple habitable patches, each of which contains two or five parapatric habitats (Fig. 1). There are five types of habitats ($H_{1, 2, \dots 5}$) in which growth performance is maximized with habitat-specific optimal trait values x_{opt_H} (x_{opt_H} = 0, 5, 10, -5, -10 for $H = H_{1, 2, \dots 5}$).

The growth performance P_i of an individual i in a habitat H_i is given by $exp\{-\varphi(x_i - x_{opt\_Hi})^2\}$, where φ is a parameter controlling the strength of natural selection. The growth performance P_i has two-fold effects on survival fitness. First, we assumed that individuals with higher growth performances (locally better-adapted individuals) have stronger competitive influence on other individuals and higher tolerance to competition. Additionally, second, we assumed that basal survival rate in the absence of competition increases proportional to the growth performance. Owing to the latter assumption, colonization of an unoccupied habitat required a certain degree of phenotypic specialization to the habitat unless natural selection is weak. A previous simulation study suggested that the effect of hybridization to promote adaptive radiation will be most pronounced under such conditions (i.e. when optimal phenotypes of distinct ecological niches are isolated by fitness valleys of maladaptive intermediate phenotypes and thus invasion of novel ecological niches requires discontinuous phenotypic change towards a novel optimal value). Such a situation would be realistic when a lineage adaptively radiates into novel ecological niches that are highly dissimilar to each other. Following these assumptions, we assumed that survival probability of an individual i is given by $w_i = P_i/\left\{1 + \left(\sum_{j=0}^{M_{Hi}} P_j/K_{Hi}(P_i)\right)\right\}$, where M_{Hi} is the number of individuals in the habitat H_i. In the absence of competitors (M_{Hi} = 0), the denominator becomes 1 and w_i equals to the growth performance P_i. Negative effect of competition on survival rate increases with the sum of growth performance of all competitors and decreases with $K_{Hi}(P_i)$, which is the tolerance of the individual i to competition. We assumed $K_{Hi}(P_i) = P_iNf/(f - 2)$ so that the tolerance to competition linearly increases with growth performance. Parameter f is per-capita female fecundity ($f > 2$) and N is a constant that determines the carrying capacity. When all individuals have the optimal trait value (and thus $P_i = 1$), carrying capacity for adults in a habitat becomes equal to N, because the demographic dynamics within a habitat approaches the classical Beverton–Holt model with carrying capacity N (for adult) when $P_i = 1$. Then, each surviving female selects a mating partner randomly from males living in the same habitat of the same patch. Females mates only once, whereas males can mate multiple times. The number of offspring per mating pair follows Po (f). Sex of newborn individuals is randomly assigned. Newborn individuals move to a neighboring patch with probability m_P and to a randomly selected habitat in the same patch with probability m_H.

Appendix S2. *Conditions under which hybridization promotes adaptive radiation*

Since we aimed to simulate repeated occurrence of hybridization-enabled adaptive radiation, we intended to use parameter sets with which hybridization is essential for rapid adaptive radiation. Therefore, prior to investigating repeated occurrence of adaptive radiations, we explored conditions under which hybridization promotes a single episode of adaptive radiation using simulations with a

single patch including five different habitats ($H_{1,2, ...,5}$). In this simulation, we allowed spontaneous mutations both before and after hybridization; thus, both spontaneous mutations and hybridization-induced genetic variation can contribute to adaptive radiation. Each simulation was started by introducing 100 clone individuals of each two allopatrically evolved lineages into the patch (see *Simulation of hybridization* in Methods for more details) and was continued for 100,000 generations after the colonization event. We performed simulations systematically varying the strength of natural selection in each habitat φ and the length of the period where two parental lineages had independently evolved in allopatry before they hybridized (T_0). To prepare pairs of two parental lineages with various values of T_0, we conducted 30 simulation replications for allopatric evolution of two populations for 200K generations, recording genotypes of all individuals at generations 0, 10K, 30K, 50K, 100K, 150K, and 200K. We used recorded populations of different time points as parental lineages for simulations of hybridization with various values of T_0. When $T_0 = 0$, there is no genetic differentiation between two parental populations, which is equivalent to the case without hybridization. With all parameter combinations that we explored, rapid adaptive radiation within 5000 generations was extremely unlikely to occur when hybridizing lineages were not genetically differentiated (Fig. S2 a-c; $T_0 = 0$), which is in accord with the results of Kagawa & Takimoto (2018). In contrast, adaptive radiation occurred within 5000 generations when hybridizing lineages were highly genetically differentiated (Fig. S2 a-c; large values of T_0). With parameter conditions that were not conducive to adaptive radiation within 5000 generations, species diversification was rarely observed even after 100,000 generations (Fig. S2 d-f). These results confirmed that adaptive radiation by spontaneous mutations alone without hybridization between genetically differentiated lineages was very unlikely with parameter values that we used. Adaptive radiation was not likely to start without hybridization producing initial standing genetic variation in our model, because we assumed that ecological niches of distinct habitats were discrete and that phenotypic effects of single mutations were not very large. As ecological niches were discrete, establishment of a population in a novel empty habitat, which is a prerequisite for natural selection to operate to cause local adaptation, required a certain level of phenotypic specialization to the habitat unless stabilizing natural selection in each habitat was extremely weak. Such phenotypes specializing to a novel habitat was not likely to evolve by accumulation of spontaneous mutations with small effects in populations inhabiting different habitats. Accordingly, standing genetic variation, that hybridization between genetically differentiated lineages can efficiently generate, played key roles for promoting colonization of new habitats in the early stage of adaptive radiation. Kagawa & Takimoto (2018) provides more comprehensive comparisons between cases with and without hybridization regarding the likelihood of adaptive radiation in face of an ecological opportunity.

Appendix S3. *Empirical basis of the default parameter values*

Default values for most of model parameters were determined as in Kagawa & Takimoto (2018). The number and length of chromosomes (n and l) and the mutation rate per locus (μ) were set to n = 15, l = 2×10^8 base-pairs (bps), and $\mu = 10^{-5}$/generation, respectively, so that they fall in realistic ranges in animals and plants. Recombination rate r was set to 10^{-8}/bp/generation, because 100 centi-Morgan typically corresponds to about 10^8 base-pairs in human genome (Lynch & Walsh 1998). The length of single loci was set to 5000 bps to be in the range of typical values (Rafalski & Morgante 2004) and to be consistent with the fact that mutation rate per locus is 100 to 10000 times higher than the mutation rate per base-pair (Lynch & Walsh 1998). For the number of loci that influence a quantitative trait, we used L = 400 instead of larger values (values in between 1000 and 10000) used in Kagawa & Takimoto (2018). Although the ratio of typical mutation rate per quantitative trait (10^{-1}/generation) and per locus ($10^{-6} \sim 10^{-5}$/generation) indicates that each trait should be influenced by more than 10,000 potential loci (Lynch & Walsh 1998), this may include many loci providing solely deleterious mutations with no contribution on adaptation. On the other hand, recent GWAS studies in human have detected 543 loci contributing variation in adult height among 458,927 individuals (Marouli *et al.* 2017), although this should overlook loci with no variation among sampled individuals. Given that human adult height is thought to be an extremely polygenic trait, we speculated that L is probably smaller than one thousand for most quantitative traits. For the default value of the phenotypic effect size of single mutations (σ_m = 0.1), we selected a small value compared to the distance between optimal values of different environments (x_{opt_H} = 0, 5, 10, 15, 20). For the default strength of natural selection within habitats (φ_B = 0.05) we choose a sufficiently high value to cause ecological speciation between populations specializing different habitats. Additionally, we performed simulations varying φ_B for the temporally repeated AR scenario. Values of the basal adult population size (N_B) and the female fecundity (f) were selected so that population size in a single habitat becomes sufficiently large to have small effect of genetic drift but not too large for simulating with our limited computational power. For default values of N_C and N_R, which control adult population size in corridor patches (in the spatially repeated AR scenario) and in the refugial phase (in the temporally repeated AR scenario), we selected values smaller than N_B but not too small to maintain a population in single habitats. Default values for the migration rate and the length of corridor in the spatially repeated AR scenario (m_P = 0.02, k = 12) were chosen to simulate long-distant range expansion that takes many generations. We also performed simulations varying k. In the temporally repeated AR scenario, we set the default migration rate between sub-populations during the refugial phase (m_R) to 0 and performed simulations varying the value of m_R. The length of allopatric evolution of parental lineages controls the amount of genetic variation generated through hybridization (Kagawa & Takimoto 2018). We selected T_0 = 200,000 as the default value so that hybridization can produce sufficiently large genetic/phenotypic variation to cause adaptive radiation (Fig. S2).

Appendix S4. *Methods to count the number of incipient species and genotypically distinct clusters of individuals*

Method to count the number of incipient species

We counted the number of genetically isolated and phenotypically distinct incipient species by using the method proposed in the Appendix S4 of Kagawa & Takimoto (2018). In order to apply the method, we recorded trait values of all individuals who had survived to maturation in two successive generations g and $g + 1$. We call individuals from the generation g + 1 "offspring-individuals". To count the number of species that exist in a focal patch, we sorted all individuals in the patch into 10 clusters based on phenotypic similarity using k-means algorithm. Then, we merged clusters that are connected by gene flow. The strength of gene flow was measured based on records of parent-offspring relationships in the two successive generations. Clusters i and j were merged when: more than t % of parents of offspring-individuals from the cluster-i belonged to another cluster j, and more than t % of parents of offspring-individuals from the cluster-j belonged to the cluster i. Additionally, if more than 50% of parents of offspring-individuals from the cluster-i belonged to another cluster j, cluster i was merged into the cluster j. This procedure was repeated until merging of clusters stopped. Finally, each cluster was counted as a species only if more than 90% of parents of offspring-individuals in the cluster belonged to the same cluster, because otherwise the cluster is likely to be a temporal hybrid swarm. Also, clusters that contained less than 20 individuals were not counted as species because such small clusters are probably ephemeral. For the threshold level of gene flow to merge clusters, we used t = 2%. With this threshold value, ecological differentiation between populations in parapatric habitats was necessary for the splitting of species. In our model, newborn individuals migrate to a randomly selected habitat at a rate 0.2 every generation. Thus, in patches with five parapatric habitats, about 4% of individuals in a habitat should be immigrants from another certain habitat when population sizes in all habitats are equivalent. Therefore, spatial isolation between parapatric habitats is not enough for splitting species under our criterion. Ecological differentiation between populations could enable speciation by strengthening natural selection against immigrants in their non-native habitats (i.e. immigrant inviability), which operates as a barrier to gene flow. We note that our definition of incipient species does not indicate good species with permanent reproductive isolation. Since our model does not incorporate non-random mating and post-zygotic reproductive isolation, incipient ecological speciation of our model can collapse if spatial isolation between habitats or divergent natural selection are removed.

Method to count the number of genotypically distinct clusters of individuals

For the temporally repeated AR scenario, we counted the number of genotypically distinct clusters of individuals at the end of simulation (after 30000 generations from the hybridization). We analyzed a subset of the population consisting of 100 randomly sampled individuals to reduce the computational

time. To analyze genotypic differences among individuals, we listed all polymorphic nucleotides among genomes of all sampled individuals. For each polymorphic nucleotide, genotype was expressed by the number of ancestral and derived nucleotide (0 and 2 indicate homozygotes of ancestral and derived nucleotide whereas 1 indicates heterozygote). Genome-wide genotype of each individual was expressed as a vector of which i-th element represents the number of ancestral and derived alleles at the i-th polymorphic nucleotide. We applied hierarchical clustering of individuals based on Euclidian distance between genome-wide genotype vectors. The number of genotypically distinct clusters of individuals was examined by counting clusters that are distant from each other by at least the threshold value D_T. Since we aimed to find genetically distinct populations rather than the statistically optimal clustering, we used an arbitrary constant threshold value $D_T = 30$, which roughly captured the number of genetically distinct populations ignoring small-scale population genetic structures generated by a few allelic polymorphisms. For the spatially repeated AR scenario, we counted the number of genotypically distinct clusters of individuals that were found in (i) only region 2 and (ii) both region 1 and 2 at the end of simulation (after 5000 generations from the hybridization). In this purpose, we first sampled 100 individuals randomly from each of region 1 and 2. Using the method described above, we identified genotypically distinct clusters in all 200 individuals sampled. Then, we counted the number of clusters that contain (i) only individuals from the region 2 and (ii) individuals from both regions. The algorithms to count genotypic clusters were implemented with the R language.

Appendix S5. *A review of potential examples where the maintenance of hybridization-induced evolvability by temporal isolation and subsequent admixture of sub-lineages might have promoted recurrent adaptive radiation.*

Hybridization generating high evolvability, followed by the maintenance of elevated evolvability by temporal isolation and secondary admixture of sub-lineages could have contributed to the repeated occurrence of adaptive radiations in archipelagos, such as Hawaiian, Galapagos, and Canary Islands. Colonization of oceanic island from mainland will induce strong population bottleneck causing the lack of genetic variation in the island population. Thus, inter- and intra-specific hybridization prior to or subsequent to the colonization of island is thought to be an important source of genetic variation for adaptive radiations in islands (Grant & Grant 2008; Baldwin & Wagner 2010; Gillespie 2016; Caujapé-Castells *et al.* 2017; Osborne *et al.* 2019). After the initial colonization of archipelago, progressive colonization of newly emerged islands sometimes leads to multiple adaptive radiations of same clades in isolated islands and at different timepoints (Stacy *et al.* 2014; Gillespie 2016). Additionally, natural disturbances, such as volcanic eruption, storms, and see-level fluctuations, could occasionally cause a collapse of island biota, followed by re-colonization and a renewal of biota (Price & Clague 2002; Schneider *et al.* 2005). These characteristics of archipelago would be conducive to repeated occurrence of adaptive radiation if clades do not lose evolutionary potential in the course of

adaptative evolution and progressive colonization of islands. Long-term observational studies of Galapagos finches suggest that temporal isolation and subsequent admixture of populations contributes to prevent the exhausting of standing genetic variation in local populations adapting to changing environment (Grant & Grant 2008). Moreover, in some other island taxa, populations in new islands show higher standing genetic variation compared to populations of their relatives in older islands, due to recent secondary hybridization events (Stacy *et al.* 2014; De Busschere *et al.* 2015; Hendrickx *et al.* 2015). Taken together, the combination of a hybridization prior to or subsequent to the initial colonization and following temporal isolation and subsequent admixture of sub-lineages may likely to promote the repeated occurrence of adaptive radiation in archipelagos. In line with this view, evidence of ancient and recent interspecific gene flow as well as instances of hybrid speciation have been found in many adaptive radiations in archipelago (Francisco-Ortega *et al.* 1996; Howarth & Baum 2005; Fjellheim *et al.* 2009; Gruenstaeudl *et al.* 2013; Papadopulos *et al.* 2013; Caujapé-Castells *et al.* 2017; Curto *et al.* 2017; White *et al.* 2018; Lamichhaney *et al.* 2018; Grant & Grant 2019; Paetzold *et al.* 2019).

Similarly, the combination of pre-existing standing genetic variation, its maintenance by temporal isolation of sub-lineages, and secondary hybridization of sub-lineages reestablishing old genetic variation might have promoted the repeated occurrence of adaptive radiations in high altitudinal mountains. For instances, recently uplifted Andes mountains and Qinghai-Tibetan plateau/Himalaya-Hengduan mountains region harbor many spectacular in-situ adaptive radiations (Drummond *et al.* 2012; Wen *et al.* 2014; Hughes & Atchison 2015; Schwery *et al.* 2015; Nevado *et al.* 2016; Diazgranados & Barber 2017; Ebersbach *et al.* 2017; Pérez-Escobar *et al.* 2017; Xing & Ree 2017; Pouchon *et al.* 2018). Geographic newness of these mountain habitats (Wen *et al.* 2014; Hughes & Atchison 2015) implies that adaptive radiations within these regions should have been rapidly established after colonization, probably with the aid of pre-existing or hybridization-induced standing genetic variation of founder populations (Pease *et al.* 2016). Biota in these mountains have been strongly affected by Tertiary and Quaternary climate oscillations. The cyclic drastic changes of temperature and moisture have caused repeated refugial isolation of lineages followed by range expansion and secondly contact (Wang *et al.* 2009; Wen *et al.* 2014; Nevado *et al.* 2018; Rangel *et al.* 2018). In fact, evidence of past interspecific gene flow and hybrid speciation has been found in many mountain radiations (Wen *et al.* 2014; Pease *et al.* 2016; Vargas *et al.* 2017; Nevado *et al.* 2018; Pouchon *et al.* 2018; Yang *et al.* 2019). The climate oscillation has also caused repeated emergence and loss of ecological opportunities (Wen *et al.* 2014), which might have caused both mass extinction and adaptive radiation (Rangel *et al.* 2018). Taken together, temporal isolation and secondary admixture of sub-lineages driven by climate cycles may have contributed to maintain pre-existing standing genetic variation during a cycle of extinction and radiation in mountain regions.

In addition to facilitating repeated occurrence of adaptive radiations, temporal isolation and

secondary admixture of lineages may also facilitate large-scale range expansion of clades and concomitant adaptive radiation into various local environments. Generally, standing genetic variation declines during range expansion (Le Corre & Kremer 1998; Excoffier *et al.* 2009; Slatkin & Excoffier 2012; Rius & Darling 2014; Dlugosch *et al.* 2015; Pfennig *et al.* 2016) (Figs. 2a, 3a), impeding both local adaptation in newly colonized areas and further range expansion. Nonetheless, there are some examples of continental-scale range expansion accompanied by adaptive radiation into various local environments (Fior *et al.* 2013; Liu *et al.* 2017; Filiault *et al.* 2018). Such radiations often involve invasion of harsh environments such as alpine habitats. Temporal isolation and secondary admixture of sub-lineages during range expansion of a lineage will promote the spread of genetic variation from the original source population to newly colonized areas. Spatial spread of genetic variation would be especially important for invasion of harsh environments where only individuals specializing to the environment to some extent can successfully establish a population (i.e. cases that specialization in advance of local adaptation is necessary for the colonization).

Supplementary references

Baldwin, B.G. & Wagner, W.L. (2010). Hawaiian angiosperm radiations of North American origin. *Ann. Bot.*, 105, 849–879.

De Busschere, C., Van Belleghem, S.M. & Hendrickx, F. (2015). Inter and intra island introgression in a wolf spider radiation from the Galápagos, and its implications for parallel evolution. *Mol. Phylogenet. Evol.*, 84, 73–84.

Caujapé-Castells, J., García-Verdugo, C., Marrero-Rodríguez, Á., Fernández-Palacios, J.M., Crawford, D.J. & Mort, M.E. (2017). Island ontogenies, syngameons, and the origins and evolution of genetic diversity in the Canarian endemic flora. *Perspect. Plant Ecol. Evol. Syst.*, 27, 9–22.

Le Corre, V. & Kremer, A. (1998). Cumulative effects of founding events during colonisation on genetic diversity and differentiation in an island and stepping-stone model. *J. Evol. Biol.*, 11, 495–512.

Curto, M., Puppo, P., Kratschmer, S. & Meimberg, H. (2017). Genetic diversity and differentiation patterns in Micromeria from the Canary Islands are congruent with multiple colonization dynamics and the establishment of species syngameons. *BMC Evol. Biol.*, 17, 198.

Diazgranados, M. & Barber, J.C. (2017). Geography shapes the phylogeny of frailejones (Espeletiinae Cuatrec., Asteraceae): a remarkable example of recent rapid radiation in sky islands. *PeerJ*, 5, e2968.

Dlugosch, K.M., Anderson, S.R., Braasch, J., Cang, F.A. & Gillette, H.D. (2015). The devil is in the details: genetic variation in introduced populations and its contributions to invasion. *Mol. Ecol.*, 24, 2095–2111.

Drummond, C.S., Eastwood, R.J., Miotto, S.T.S. & Hughes, C.E. (2012). Multiple Continental Radiations

and Correlates of Diversification in Lupinus (Leguminosae): Testing for Key Innovation with Incomplete Taxon Sampling. *Syst. Biol.*, 61, 443–460.

Ebersbach, J., Schnitzler, J., Favre, A. & Muellner-Riehl, A.N. (2017). Evolutionary radiations in the species-rich mountain genus Saxifraga L. *BMC Evol. Biol.*, 17, 119.

Excoffier, L., Foll, M. & Petit, R.J. (2009). Genetic Consequences of Range Expansions. *Annu. Rev. Ecol. Evol. Syst.*, 40, 481–501.

Filiault, D.L., Ballerini, E.S., Mandáková, T., Aköz, G., Derieg, N.J., Schmutz, J., *et al.* (2018). The Aquilegia genome provides insight into adaptive radiation and reveals an extraordinarily polymorphic chromosome with a unique history. *Elife*, 7, 1–31.

Fior, S., Li, M., Oxelman, B., Viola, R., Hodges, S.A., Ometto, L., *et al.* (2013). Spatiotemporal reconstruction of the Aquilegia rapid radiation through next-generation sequencing of rapidly evolving cpDNA regions. *New Phytol.*, 198, 579–592.

Fjellheim, S., Jørgensen, M.H., Kjos, M. & Borgen, L. (2009). A molecular study of hybridization and homoploid hybrid speciation in Argyranthemum (Asteraceae) on Tenerife, the Canary Islands. *Bot. J. Linn. Soc.*, 159, 19–31.

Francisco-Ortega, J., Jansen, R.K. & Santos-Guerra, A. (1996). Chloroplast DNA evidence of colonization, adaptive radiation, and hybridization in the evolution of the Macaronesian flora. *Proc. Natl. Acad. Sci.*, 93, 4085–4090.

Gillespie, R.G. (2016). Island time and the interplay between ecology and evolution in species diversification. *Evol. Appl.*, 9, 53–73.

Grant, B.R. & Grant, P.R. (2008). Fission and fusion of Darwin's finches populations. *Philos. Trans. R. Soc. B Biol. Sci.*, 363, 2821–2829.

Grant, P.R. & Grant, B.R. (2019). Hybridization increases population variation during adaptive radiation. *Proc. Natl. Acad. Sci.*, 116, 23216–23224.

Gruenstaeudl, M., Santos-Guerra, A. & Jansen, R.K. (2013). Phylogenetic analyses of Tolpis Adans. (Asteraceae) reveal patterns of adaptive radiation, multiple colonization and interspecific hybridization. *Cladistics*, 29, 416–434.

Hendrickx, F., Backeljau, T., Dekoninck, W., Van Belleghem, S.M., Vandomme, V. & Vangestel, C. (2015). Persistent inter- and intraspecific gene exchange within a parallel radiation of caterpillar hunter beetles (Calosoma sp.) from the Galápagos. *Mol. Ecol.*, 24, 3107–3121.

Howarth, D.G. & Baum, D.A. (2005). Genealogical evidence of homoploid hybrid speciation in an adaptive radiation of Scaevola (Goodeniaceae) in the Hawaiian Islands. *Evolution (N. Y).*, 59, 948–961.

Hughes, C.E. & Atchison, G.W. (2015). The ubiquity of alpine plant radiations: from the Andes to the Hengduan Mountains. *New Phytol.*, 207, 275–282.

Kagawa, K. & Takimoto, G. (2018). Hybridization can promote adaptive radiation by means of

transgressive segregation. *Ecol. Lett.*, 21, 264–274.

Lamichhaney, S., Han, F., Webster, M.T., Andersson, L., Grant, B.R. & Grant, P.R. (2018). Rapid hybrid speciation in Darwin’s finches. *Science (80-.).*, 359, 224–228.

Liu, Y., Li, D., Zhang, Q., Song, C., Zhong, C., Zhang, X., *et al.* (2017). Rapid radiations of both kiwifruit hybrid lineages and their parents shed light on a two-layer mode of species diversification. *New Phytol.*, 215, 877–890.

Lynch, M. & Walsh, B. (1998). *Genetics and analysis of quantitative traits*. Sinauer, Sunderland, MA.

Marouli, E., Graff, M., Medina-Gomez, C., Lo, K.S., Wood, A.R., Kjaer, T.R., *et al.* (2017). Rare and low-frequency coding variants alter human adult height. *Nature*, 542, 186–190.

Nevado, B., Atchison, G.W., Hughes, C.E. & Filatov, D.A. (2016). Widespread adaptive evolution during repeated evolutionary radiations in New World lupins. *Nat. Commun.*, 7, 12384.

Nevado, B., Contreras-Ortiz, N., Hughes, C. & Filatov, D.A. (2018). Pleistocene glacial cycles drive isolation, gene flow and speciation in the high-elevation Andes. *New Phytol.*, 219, 779–793.

Osborne, O.G., Ciezarek, A., Wilson, T., Crayn, D., Hutton, I., Baker, W.J., *et al.* (2019). Speciation in Howea Palms Occurred in Sympatry, Was Preceded by Ancestral Admixture, and Was Associated with Edaphic and Phenological Adaptation. *Mol. Biol. Evol.*, 1–16.

Paetzold, C., Wood, K.R., Eaton, D.A.R., Wagner, W.L. & Appelhans, M.S. (2019). Phylogeny of Hawaiian Melicope (Rutaceae): RAD-seq Resolves Species Relationships and Reveals Ancient Introgression. *Front. Plant Sci.*, 10, 1–16.

Papadopulos, A.S.T., Price, Z., Devaux, C., Hipperson, H., Smadja, C.M., Hutton, I., *et al.* (2013). A comparative analysis of the mechanisms underlying speciation on Lord Howe Island. *J. Evol. Biol.*, 26, 733–745.

Pease, J.B., Haak, D.C., Hahn, M.W. & Moyle, L.C. (2016). Phylogenomics Reveals Three Sources of Adaptive Variation during a Rapid Radiation. *PLOS Biol.*, 14, e1002379.

Pérez-Escobar, O.A., Chomicki, G., Condamine, F.L., Karremans, A.P., Bogarín, D., Matzke, N.J., *et al.* (2017). Recent origin and rapid speciation of Neotropical orchids in the world’s richest plant biodiversity hotspot. *New Phytol.*, 215, 891–905.

Pfennig, K.S., Kelly, A.L. & Pierce, A.A. (2016). Hybridization as a facilitator of species range expansion. *Proc. R. Soc. B Biol. Sci.*, 283, 20161329.

Pouchon, C., Fernández, A., Nassar, J.M., Boyer, F., Aubert, S., Lavergne, S., *et al.* (2018). Phylogenomic Analysis of the Explosive Adaptive Radiation of the Espeletia Complex (Asteraceae) in the Tropical Andes. *Syst. Biol.*, 67, 1041–1060.

Price, J.P. & Clague, D.A. (2002). How old is the Hawaiian biota? Geology and phylogeny suggest recent divergence. *Proc. R. Soc. London. Ser. B Biol. Sci.*, 269, 2429–2435.

Rafalski, A. & Morgante, M. (2004). Corn and humans: recombination and linkage disequilibrium in two genomes of similar size. *Trends Genet.*, 20, 103–111.

Rangel, T.F., Edwards, N.R., Holden, P.B., Diniz-Filho, J.A.F., Gosling, W.D., Coelho, M.T.P., *et al.* (2018). Modeling the ecology and evolution of biodiversity: Biogeographical cradles, museums, and graves. *Science (80-.).*, 361, eaar5452.

Rius, M. & Darling, J.A. (2014). How important is intraspecific genetic admixture to the success of colonising populations? *Trends Ecol. Evol.*, 29, 233–242.

Schneider, H., Ranker, T.A., Russell, S.J., Cranfill, R., Geiger, J.M.O., Aguraiuja, R., *et al.* (2005). Origin of the endemic fern genus Diellia coincides with the renewal of Hawaiian terrestrial life in the Miocene. *Proc. R. Soc. B Biol. Sci.*, 272, 455–460.

Schwery, O., Onstein, R.E., Bouchenak-Khelladi, Y., Xing, Y., Carter, R.J. & Linder, H.P. (2015). As old as the mountains: the radiations of the Ericaceae. *New Phytol.*, 207, 355–367.

Slatkin, M. & Excoffier, L. (2012). Serial Founder Effects During Range Expansion: A Spatial Analog of Genetic Drift. *Genetics*, 191, 171–181.

Stacy, E.A., Johansen, J.B., Sakishima, T., Price, D.K. & Pillon, Y. (2014). Incipient radiation within the dominant Hawaiian tree Metrosideros polymorpha. *Heredity (Edinb).*, 113, 334–342.

Vargas, O.M., Ortiz, E.M. & Simpson, B.B. (2017). Conflicting phylogenomic signals reveal a pattern of reticulate evolution in a recent high-Andean diversification (Asteraceae: Astereae: Diplostephium). *New Phytol.*, 214, 1736–1750.

Wang, L., Abbott, R.J., Zheng, W.E.I., Chen, P., Wang, Y. & Liu, J. (2009). History and evolution of alpine plants endemic to the Qinghai-Tibetan Plateau: Aconitum gymnandrum (Ranunculaceae). *Mol. Ecol.*, 18, 709–721.

Wen, J., Zhang, J., Nie, Z., Zhong, Y. & Sun, H. (2014). Evolutionary diversifications of plants on the Qinghai-Tibetan Plateau. *Front. Genet.*, 5, 4.

White, O.W., Reyes-Betancort, A., Chapman, M.A. & Carine, M.A. (2018). Independent homoploid hybrid speciation events in the Macaronesian endemic genus Argyranthemum. *Mol. Ecol.*, 27, 4856–4874.

Xing, Y. & Ree, R.H. (2017). Uplift-driven diversification in the Hengduan Mountains, a temperate biodiversity hotspot. *Proc. Natl. Acad. Sci.*, 114, E3444–E3451.

Yang, R., Folk, R., Zhang, N. & Gong, X. (2019). Homoploid hybridization of plants in the Hengduan mountains region. *Ecol. Evol.*, 9, 8399–8410.

Table S1. Model Parameters.

Definition	Symbol	Default in spatially repeated AR	Default in temporally repeated AR	Alternative values examined
The number of loci	L	400	400	–
The number of chromosomes	n	15	15	–
The length of each chromosome	l	2×10^8	2×10^8	–
Mutation rate/locus/generation	μ	10^{-5}	10^{-5}	–
Phenotypic effect size of a mutation	σ_m	10^{-1}	10^{-1}	–
Recombination rate/generation	r	$1/10^8$bps	$1/10^8$bps	–
Female fecundity	f	10	10	–
Selection strength during allopatric evolution of parental lineages	φ_P	0.05	0.05	–
Basal selection strength	φ_B	0.05	0.05	0, 0.025, 0.1, 0.2, 0.4
Selection strength in corridor patches	φ_C	0.1	–	0, 0.025, 0.05, 0.2, 0.4
Selection strength during the refugial phase	φ_R	–	0	–
Corridor length	k	12	–	4, 8, 16, 20
Basal carrying capacity (adult population size) of single habitat	N_B	100	100	–
Carrying capacity of single habitat in corridor patch	N_C	20	–	10, 30, 40, 50
Carrying capacity of single habitat during the refugial phase	N_R	–	20	10, 40, 80, 160
Basal migration rate b/w patches	m_P	0.01	0.1	–
Migration rate b/w patches during the refugial phase	m_R	–	0	10^{-5}, 10^{-4}, 10^{-3}
Moving rate b/w habitats within patch	m_H	0.2	0.2	–
The duration of allopatric evolution of parental lineages (generations)	T_0	2×10^5	2×10^5	–

Supplementary Figures

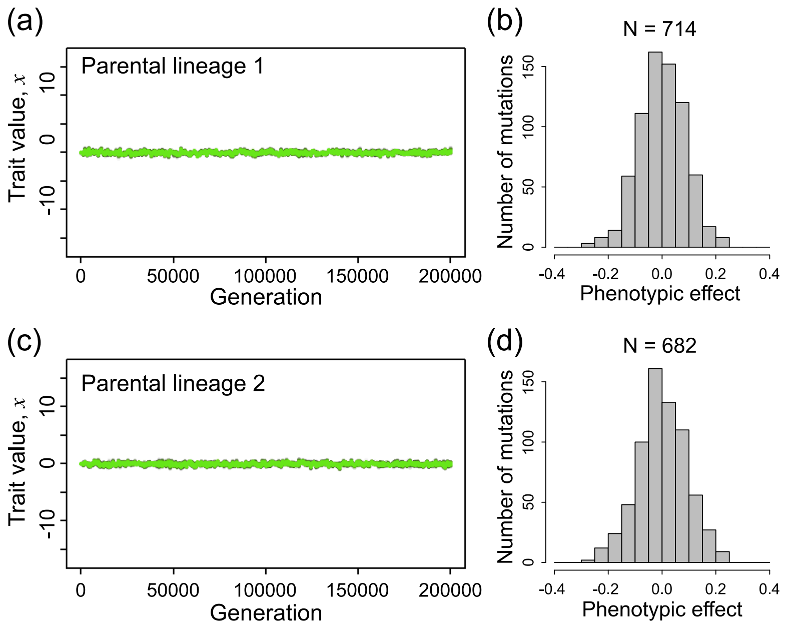

Figure S1. Example of evolutionary dynamics during allopatric evolution of parental lineages. (a), (c) Dynamics of phenotypic evolution. Trait value x of all individuals of lineages 1 and 2 are shown for each generation. Trait value x of two parental lineages fluctuated at around the optimal trait value x = 0. (b), (d) Histograms of phenotypic effects of mutations fixed in lineages 1 and 2 after 200,000 generations from their isolation. Genomes of lineages 1 and 2 have independently fixed 714 and 682 de novo mutations on 400 loci controlling the trait x, although many mutations were nearly neutral. Trait values of both lineages stayed around $x = 0$, which is the optimal value of the stabilizing selection for parental lineages. In spite of the stasis in phenotypic value, many mutations accumulated in their genome through fixation of mutations with compensating phenotypic effects (i.e. positive and negative effects).

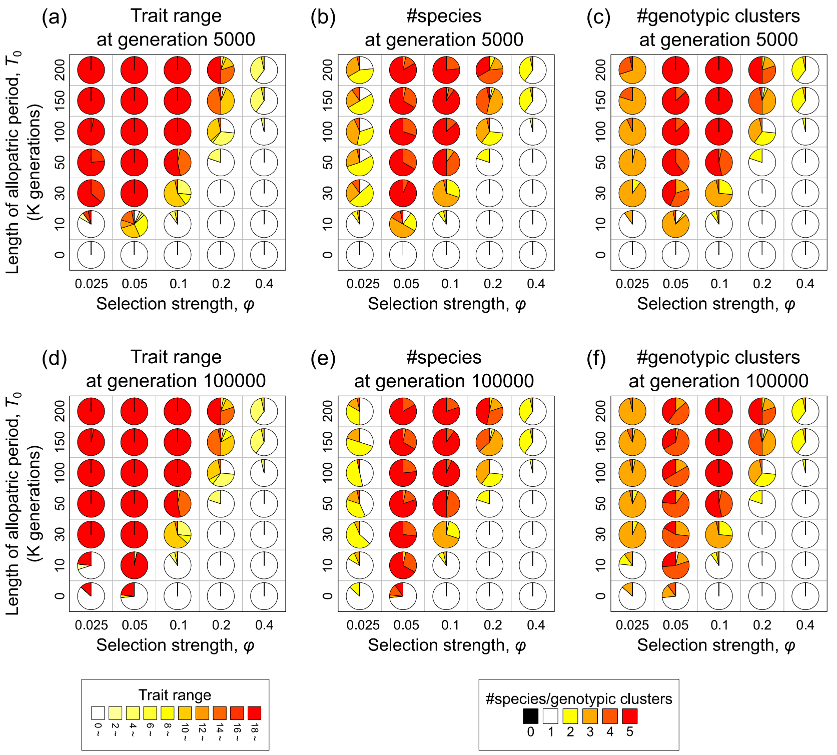

Figure S2. Conditions for single episode of adaptive radiation within a single environmentally heterogeneous region. Pie charts in each panel show frequencies of (a, d) trait range, (b, e) the number of phenotypically distinct and reproductively isolated species, and (c, f) the number of genotypically distinct clusters of individuals (N = 30 runs for each condition). Trait range was quantified by the distance between the 0.025 and 0.975 quantiles of the phenotypic distribution. Without hybridization between genetically differentiated lineages ($T_0 = 0$), rapid adaptive radiation within 5000 generations was extremely unlikely to occur ($T_0 = 0$ in panels a-c). In such cases, species diversification rarely observed even after long-term evolution from the colonization event ($T_0 = 0$ in panels d-f), especially when stabilizing natural selection within each habitat was strong ($\varphi \geq 0.1$). With hybridization between genetically differentiated lineages (large T_0), in contrast, adaptive radiation could occur within 5000 generations unless natural selection was extremely strong. Extremely strong stabilizing natural selection within each habitat made adaptive radiation difficult by impeding invasion of new unoccupied habitats. This effect was owing to our model assumption that individuals with maladaptive phenotypes in their local environment have low survival rates even in the absence of competitors. When natural selection was very weak ($\varphi = 0.025$), on the other hand, the number of species remained small despite that wide range of trait values have evolved. This was because immigrant inviability, which contributes to reduce gene flow between ecologically differentiated populations, was mitigated when stabilizing natural selection in each habitat was weak. These results confirmed that adaptive

radiation was unlikely to occur without hybridization between genetically differentiated lineages in our model with parameter values that we used.

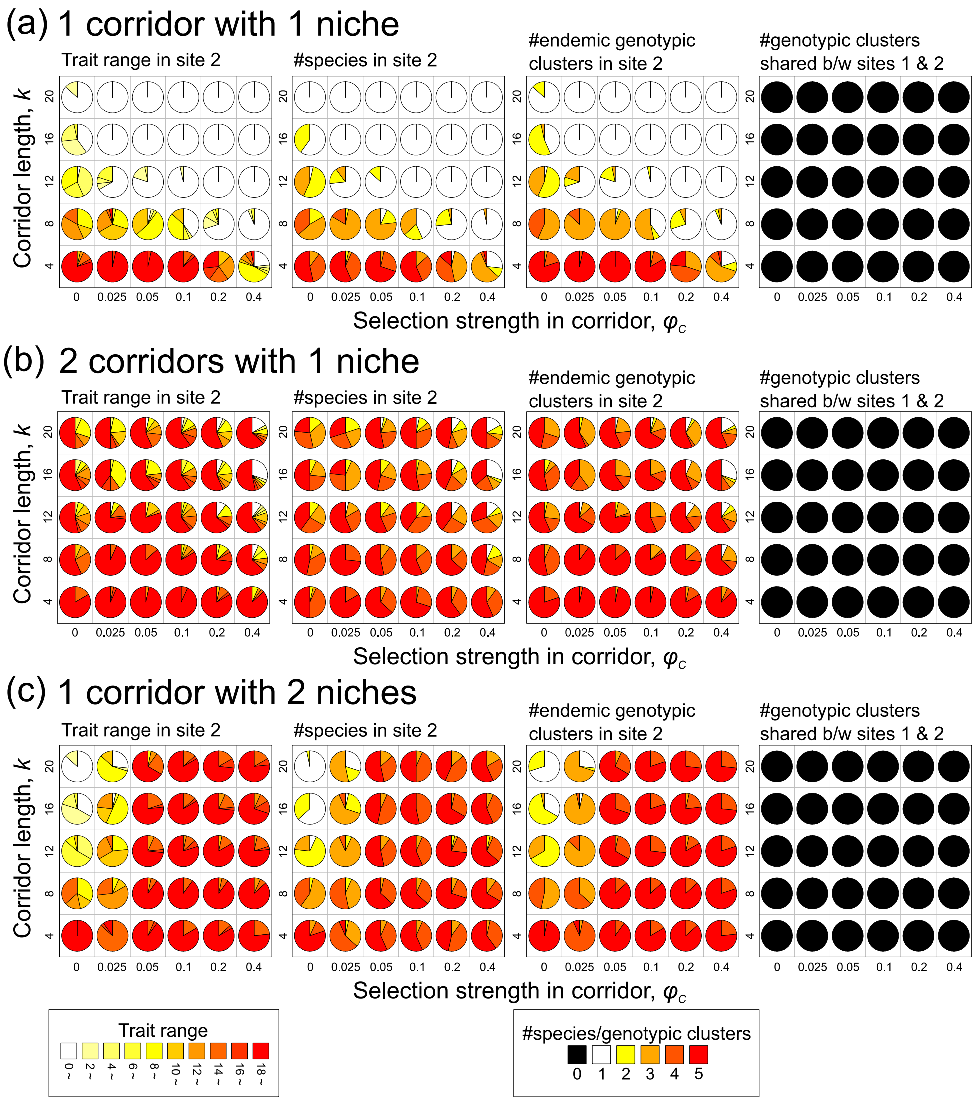

Figure S3. Detailed analysis of simulation results shown in the Fig. 3. Pie charts in each panel show frequencies of (from left to right) (1) trait range in region 2, (2) the number of reproductively isolated and phenotypically distinct species in region 2 (same as Fig. 3), (3) the number of genotypically distinct clusters of individuals that are found only in region 2, and (4) the number of genotypically distinct clusters of individuals that are found in both region 1 and 2 (N = 30 simulation runs for each condition). Trait range was quantified by the distance between the 0.025 and 0.975 quantiles of the phenotypic distribution within region 2. (a) Results with single corridor without environmental heterogeneity. (b) Results with two geographically isolated corridors without environmental heterogeneity. (c) Results with single corridor with two distinct environments. Other parameters were set to default values (Table 1). In all simulation runs, there were no genotypic clusters shared between region 1 and 2. Consistently, the number of genotypic clusters that were endemic in region 2 was equivalent to the number of reproductively isolated species in region 2. These results together imply

independent evolution of phenotypically diverse species in two regions. As speciation in our model requires ecological differentiation, the number of species in region 2 was proportional to the trait range in region 2. The scarcity of species shared between two regions is reasonable in our model for three reasons. First, as a population in corridor is established by a subset of the hybrid swarm in region 1 before the loss of hybridization-induced standing genetic variation, species in region 1 and sub-lineage(s) in corridor(s) will fix different sets of genes. Owing to this founder effect, colonizers of region 2 will be genotypically distinct from species in region 1. Although gene flow between two regions through corridor populations can homogenize genotypes of species using the habitat 1, such genotypic homogenization has not been observed within 5000 generations from hybridization. Second, when secondary admixture between the genetically isolated sub-lineages occurs in region 2, increased genotypic diversity will lead to new genotypic combinations to be recruited into the new species in region 2. Third, even when the corridor environment imposes weak or no selection and potentially allows coexistence of multiple species, direct colonization of multiple species is difficult in our model because weak selection in the corridor will weaken reproductive isolation between species such that they will merge in the course of their range expansion.

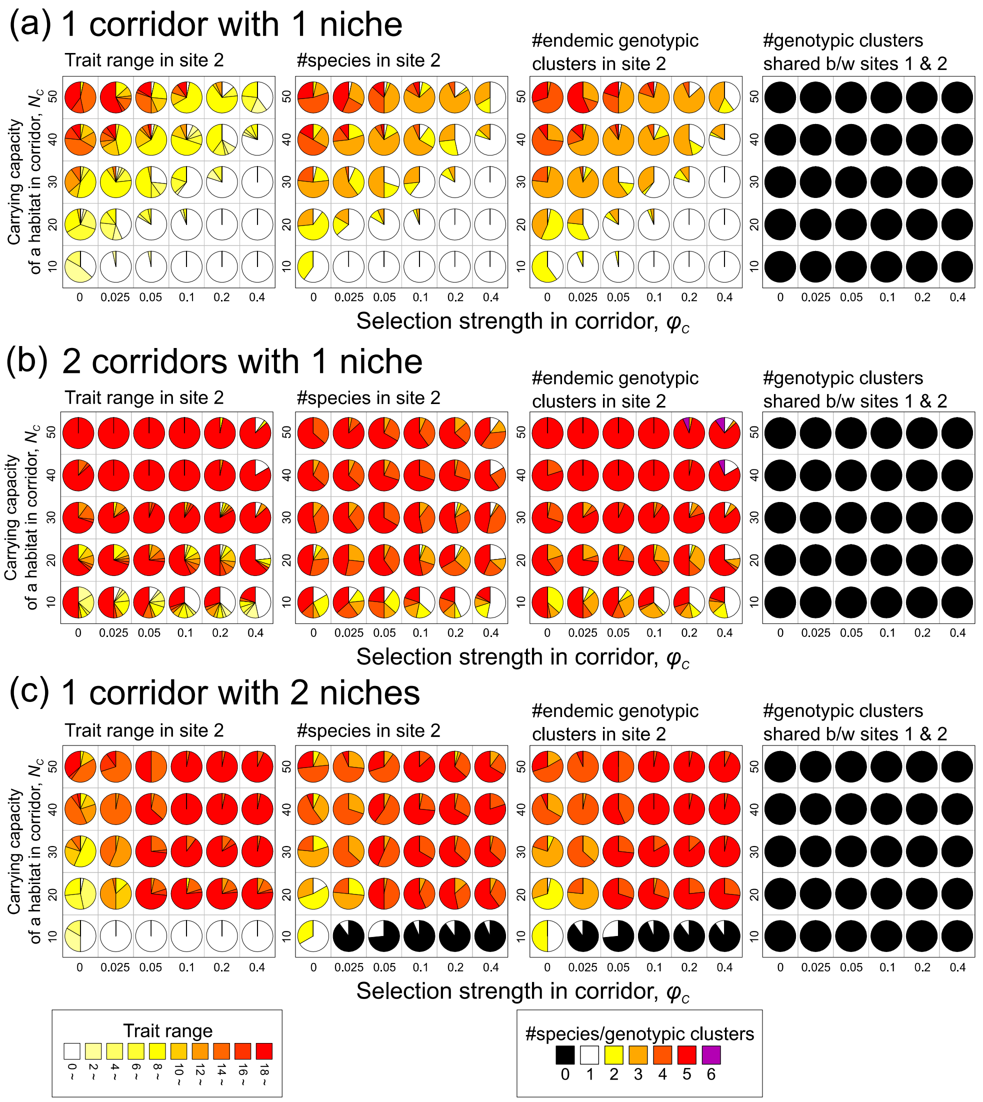

Figure S4. Effects of the carrying capacity of single habitats in corridor patch N_C, and the natural selection strength within corridor patches φ_C in the spatially repeated AR scenario. We performed simulations with (a) a single environmentally homogeneous corridor, (b) two geographically isolated environmentally homogeneous corridors, and (c) a single corridor containing habitats with two distinct environments. Other parameters were set to default values (Table 1).

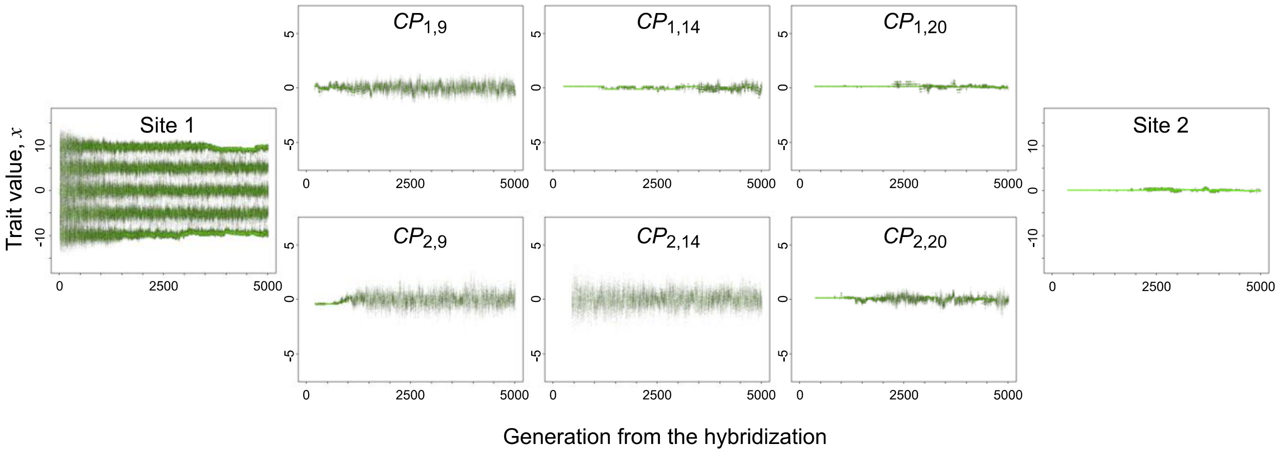

Figure S5. An example of failure of geographically repeated adaptive radiations with two geographically isolated corridors. Trait value x of all individuals in two regions and corridor patches $CP_{i,9}$, $CP_{i,14}$, and $CP_{i,20}$ (i = 1, 2) are shown for each generation. Individuals who did and did not survive to participated in reproduction are shown in green and grey respectively. In this run, spread of hybridization-induced genetic variation from region 1 to 2 was prevented probably by formation of a hybrid zone in the corridor 2. Hybrid zone could be formed on a corridor when a lineage from one corridor reaches the region 2 earlier than the lineage of the other corridor and then migration from region 2 to the other corridor leads to collision of two lineages in corridor. In this simulation example, we observed high phenotypic variation at around the corridor patch $CP_{2,14}$. As novel hybrid phenotypes are selected against in corridor patches, genetic variation of hybrids would not easily spread out from the hybrid zone. Consequently, adaptive radiation in region 2 did not occur in 5000 generations from the hybridization event. Parameter values: k = 20 and other parameters were set to default values (Table 1).

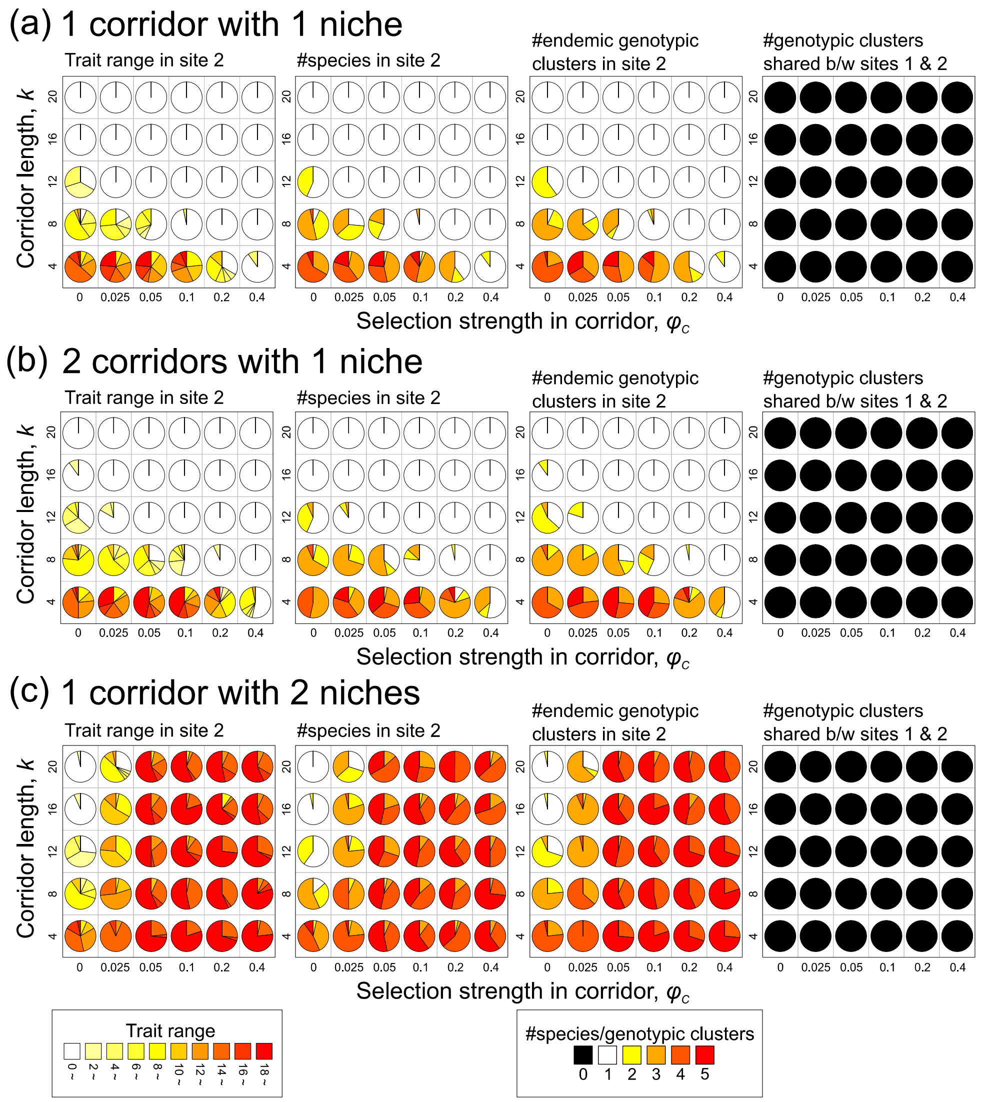

Figure S6. The spatially repeated adaptive radiation mediated by two geographically isolated corridors was diminished if there was a time lag in the introduction of two parental lineages. In this simulation, 100 individuals of parental lineages 1 and 2 were introduced to region 1 at the generation 0 and 100, respectively. We simulated cases with (a) a single environmentally homogeneous corridor, (b) two geographically isolated environmentally homogeneous corridors, and (c) a single corridor containing habitats with two distinct environments. Additionally, we systematically varied the length of corridor k, and the natural selection strength within corridor patches φ_C. Other parameters were set to default values (Table 1). Comparison of Figs 3b and S6b indicates that the time lag in introduction of two parental lineages suppresses repeated adaptive radiation mediated by two expansion corridors. In such situation, the first lineage spread across the entire system of connected patches before the introduction of the other parental lineage. Then, the arrival of the second lineage in region 1 generated a hybrid population and facilitated an adaptive radiation in region 1, which then led to formation of a hybrid-

zone between the hybrid-lineage and lineage 1 in the corridors. This impeded the spread of genes from the second parental lineage toward the second region, because most of hybrid genotypes with associated phenotypes were less well adapted to the habitat in corridor patches compared to genotypes of the resident lineage 1 population. However, our additional simulations revealed that the presence of two isolated paths for range expansion still slightly elevated the likelihood of repeated adaptive radiation in long-term even if there was a time lag in the introduction of two parental lineages (Fig. S7).

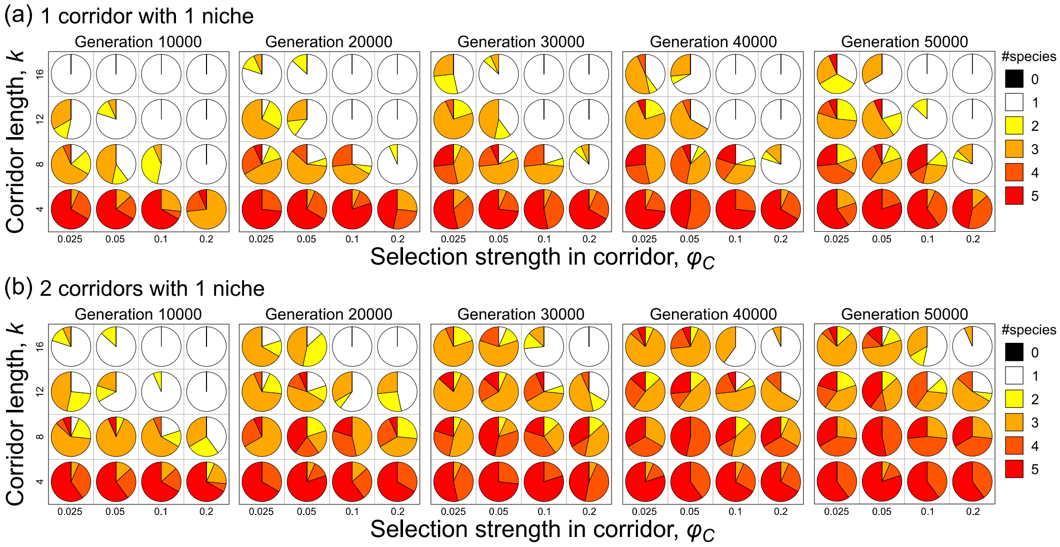

Figure S7. Long-term evolutionary effect of the presence of two independent corridors in the spatially repeated AR scenario with a time lag in introduction of two parental lineages. To investigate the long-term effect of the presence of two corridors, we performed 15 simulations runs for 50000 (instead of 5000) generations for a subset of parameter space of Fig. S6a, b. Other simulation conditions are same as in the Fig. S6a, b. (a) Results with a single environmentally homogeneous corridor. (b) Results with two geographically isolated environmentally homogeneous corridors. Panels show the number of species in region 2 at the 10000th, 20000th, 30000th, 40000th, and 50000th generations from the hybridization event. Simulation results confirmed that the feasibility of repeated adaptive radiation was higher with two geographically isolated corridors than with only a single corridor. However, the time lag in the introduction of two parental lineages delayed the start of adaptive radiation in region 2 compared to the case of simultaneous introduction of two parental lineages (Fig. 3b). Additionally, the effect of the presence of two isolated corridors to promote repeated adaptive radiation was not as strong as in the case of simultaneous introduction of two parental lineages (Fig. 3).

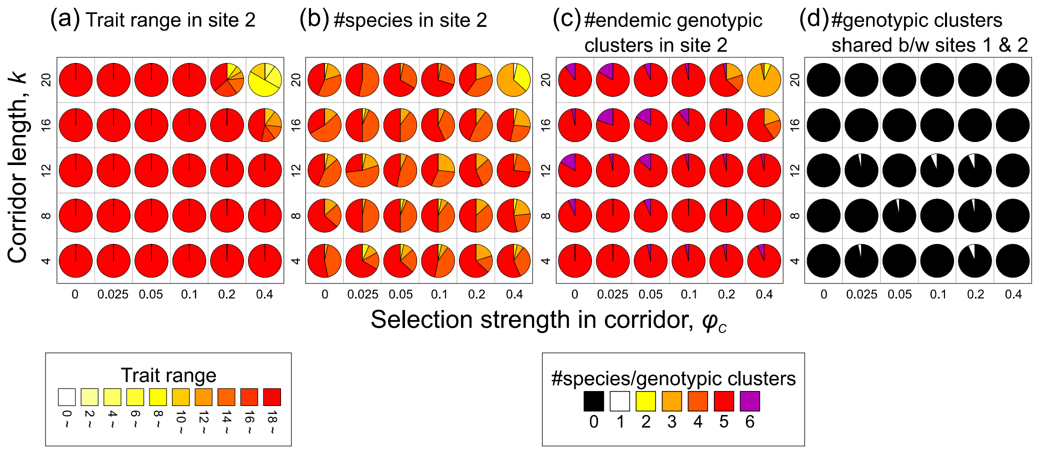

Figure S8. Effect of one-way migration in the spatially repeated AR scenario with a single environmentally homogeneous corridor. In simulations shown here, only migration in the direction from region 1 to 2 is allowed (i.e. migration from $CP_{i,j}$ to $CP_{i,j+1}$ is allowed whereas migration from $CP_{i,j}$ to $CP_{i,j-1}$ is forbidden). We performed simulations systematically varying the length of the corridor k and the strength of natural selection in corridor patches φ_C. Other parameters were set to default values (Table 1). Simulation results demonstrate that repeated adaptive radiations at geographically distant areas is possible even with a single environmentally homogeneous corridor if migration is one-way.

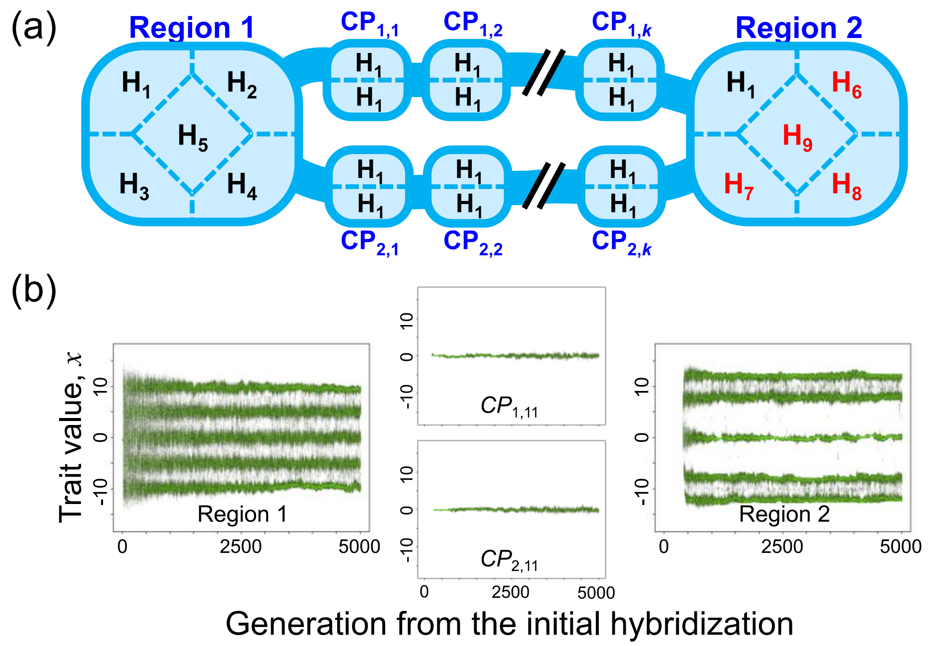

Figure S9. A simulation example with an alternative version of the spatially repeated AR scenario in which fitness optima are different between region 1 and 2. (a) Ecological and geographic condition of the simulation. The region 1 contains habitats H_1, H_2, H_3, H_4, and H_5, whereas the region 2 contains H_1, H_6, H_7, H_8, and H_9. Corridor patches contain only the habitat H_1 (i.e. environmentally homogeneous corridor). Optimal trait values of nine habitats were set as follows: $x_{opt\_H1} = 0$; $x_{opt\_H2} = 5$; $x_{opt\_H3} = 10$; $x_{opt\_H4} = -5$; $x_{opt\_H5} = -10$; $x_{opt\_H6} = 7.5$; $x_{opt\_H7} = 12.5$; $x_{opt\_H8} = -7.5$; $x_{opt\_H9} = -12.5$. The simulation assumed two geographically isolated corridors with only a single habitat type H_1. (b) Evolutionary dynamics. Parameters: corridor length $k = 20$; other parameters: default values in Table S1. In the region 2, secondary admixture of sub-lineages from two isolated corridors generated various phenotypes including those with low fitness in the region 1, which enabled rapid evolution of four incipient species specialized to novel habitats that did not exist in the region 1.

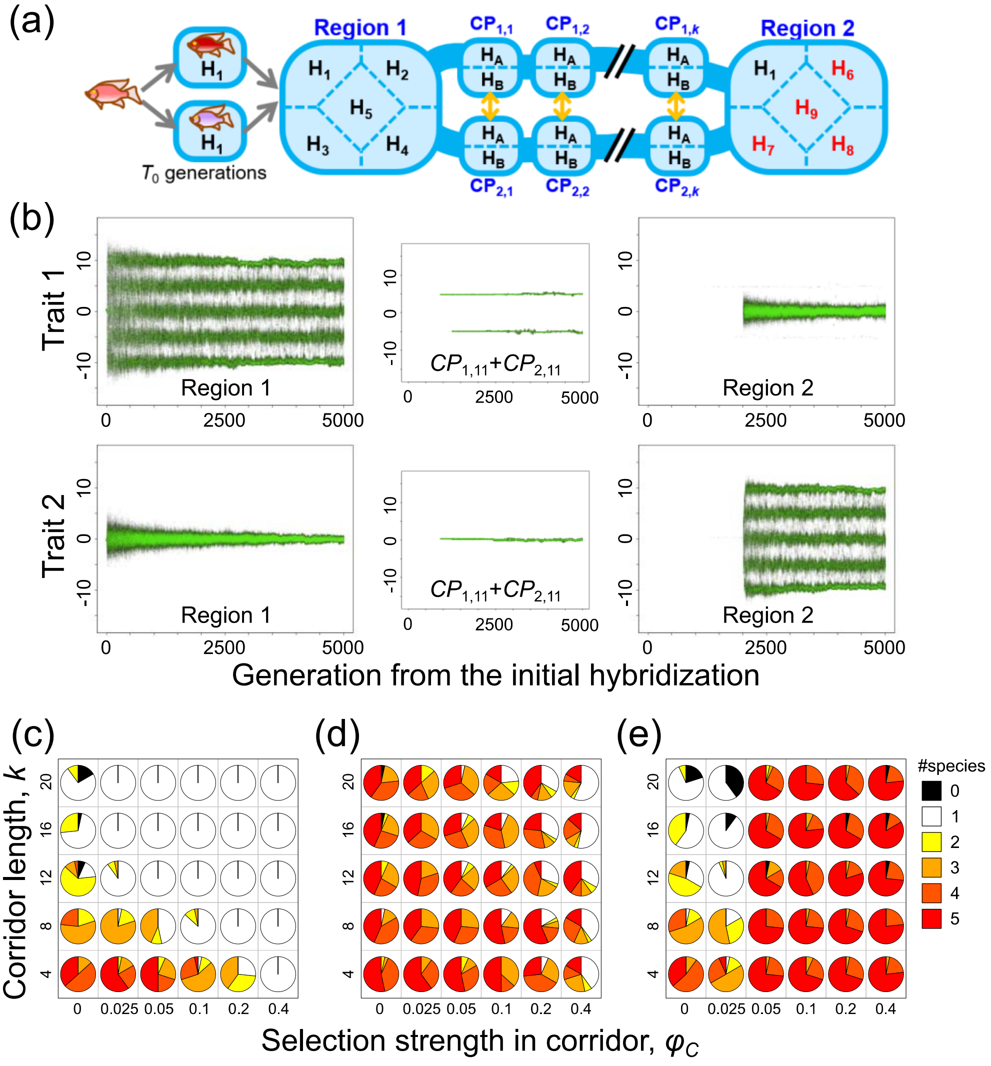

Figure S10. Simulations with two ecological traits. (a) Ecological and geographic condition of the simulation. We considered the spatially repeated AR scenario, in which only the first ecological trait is subject to divergent selection in the region 1 and only the second ecological trait is subject to divergent selection in the region 2. There are nine types of habitats $H_{1,\ 2,\ \ldots,\ 9}$. Optimal trait combinations of nine habitats were set as follows: $\boldsymbol{x}_{opt\_H1} = (0, 0)$; $\boldsymbol{x}_{opt\_H2} = (5, 0)$; $\boldsymbol{x}_{opt\_H3} = (10, 0)$; $\boldsymbol{x}_{opt\_H4} = (-5, 0)$; $\boldsymbol{x}_{opt\_H5} = (-10, 0)$; $\boldsymbol{x}_{opt\_H6} = (0, 5)$; $\boldsymbol{x}_{opt\_H7} = (0, 10)$; $\boldsymbol{x}_{opt\_H8} = (0, -5)$; $\boldsymbol{x}_{opt\_H9} = (0, -10)$. The region 1 contains habitats H_1, H_2, H_3, H_4, and H_5, whereas the region 2 contains H_1, H_6, H_7, H_8, and H_9. Each corridor patch contains two habitats H_A and H_B. We assume $H_A = H_B = H_1$ for cases with environmentally homogeneous corridor, whereas $H_A = H_2$ and $H_B = H_4$ for cases with environmentally heterogeneous corridor. Thus, the trait 2 is always subject to stabilizing selection in corridor(s). Before simulating the hybridization, we simulated evolution of two parental lineages in allopatric patches with the habitat H_1 for T_0 generations. (b) An example of simulation with single corridor with environmental heterogeneity. Two ecological traits of all individuals in region 1, 2 and the middle

patch of the corridor are shown for each generation. Parameters: corridor length k = 20; other parameters: default values in Table S1. In the region 1, a hybrid population rapidly diverged into five incipient species with distinct phenotypes of the ecological trait 1 but with the same phenotype of the ecological trait 2. Two of them expanded their range towards the region 2 through the corridor using two distinct habitats of the corridor. Subsequently, two incipient species, which were genetically isolated in region 1 and corridor by ecological natural selection on the trait 1, admixed in the region 2. This reestablished phenotypic variation in not only the trait 1 but also the trait 2 through transgressive segregation. This enabled adaptive radiation in the region 2 in response to divergent natural selection on the trait 2. This result supports that incipient speciation by ecological divergent selection on a single trait axis can maintain potential genetic variation for other trait axes subjected to stabilizing selection. Secondary admixture between such incipient species can promote rapid adaptive diversification along novel trait axes that have never diversified in the previous radiation. (c-e) Results of simulations varying the corridor length and the strength of natural selection in corridor(s). Pie charts show frequencies of the number of incipient species in the region 2 after 5000 generations from the initial hybridization event (N = 30 runs for each condition). (c) With single corridor without environmental heterogeneity. (d) With two geographically isolated corridors without environmental heterogeneity. (e) With single corridor with environmental heterogeneity. Other parameters were set to the default values in Table S1.

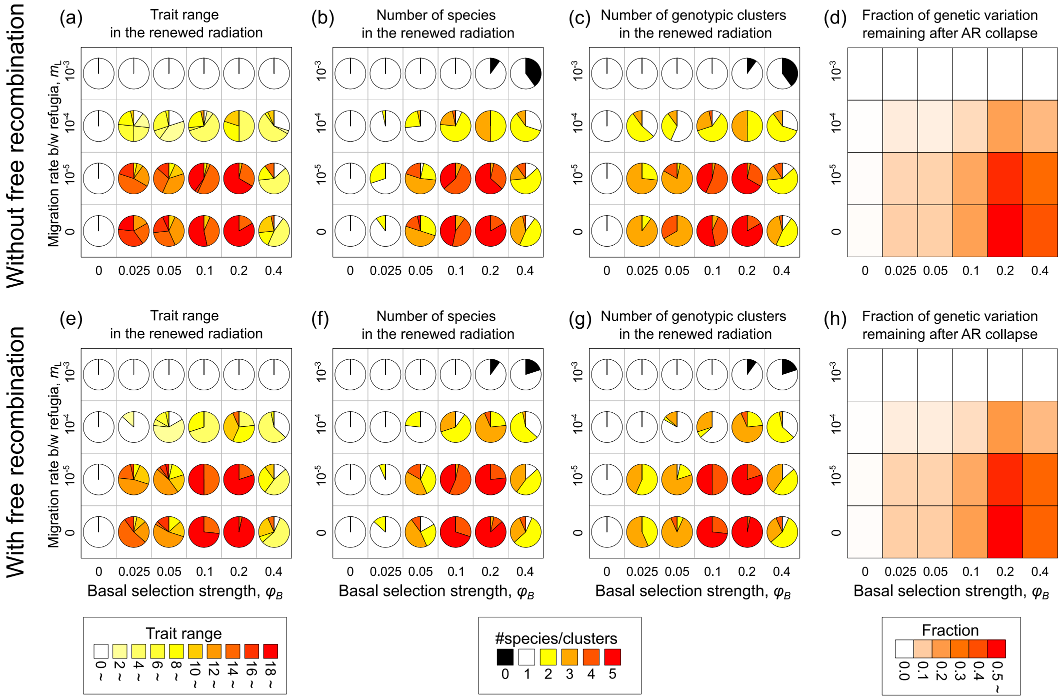

Figure S11. Results of simulations of the temporally repeated AR scenario without and with an artificial elimination of effects of genetic linkage structures which are built up before the collapse of the first adaptive radiation. We performed simulations systematically varying the basal strength of divergent selection φ_B and the migration rate between three patches during the refugial phase m_R. Other parameters were set to default values (Table 1). Pie charts show the trait range (a, e), the number of species (b, f), and the number of genotypically distinct clusters of individuals (c, g) at the generation 30000. (d, h) The extent of evolutionary potential at the end of the refugial phase (generation 20000). (a-d) Results without the artificial elimination of effects of genetic linkage structures. The data set is same as in the Fig. 5. (e-h) Results of simulations with the artificial elimination of effects of genetic linkage structures. In these simulations, we assumed free recombination between all derived nucleotides only in the refugial phase, which will completely destroy genetic linkage structures that have evolved in the first adaptive radiation. Effects of genetic linkage structures to facilitate rapid reconstruction of adaptive radiation can be evaluated by comparing results of simulations with and without the free recombination in the refugial phase. The artificial elimination of genetic linkage structures during the refugial phase slightly hindered the rapid re-evolution of adaptive radiation. This effect was most pronounced with weak basal strength of natural selection. Especially, when $\varphi_B = 0.025$ and $m_L = 0$, both the trait range and the number of genotypic clusters in the renewed radiation were statistically significantly different between sets of 30 simulation replications with and without free recombination: Mean values of the trait range with and without free recombination were 14.0 and 16.2,

respectively; $p < 10^{-4}$ with Wilcoxon rank sum test; Mean values of the number of genotypic clusters with and without free recombination were 2.57 and 2.90, respectively; p =0.00325 with Wilcoxon rank sum test with normal approximation (we cannot compute the exact p-value due to ties). This result suggests that genetic linkage structures which have been built up through adaptation have facilitated rapid re-evolution of adaptive phenotypic variation, although the effect was very slight in our simulation.

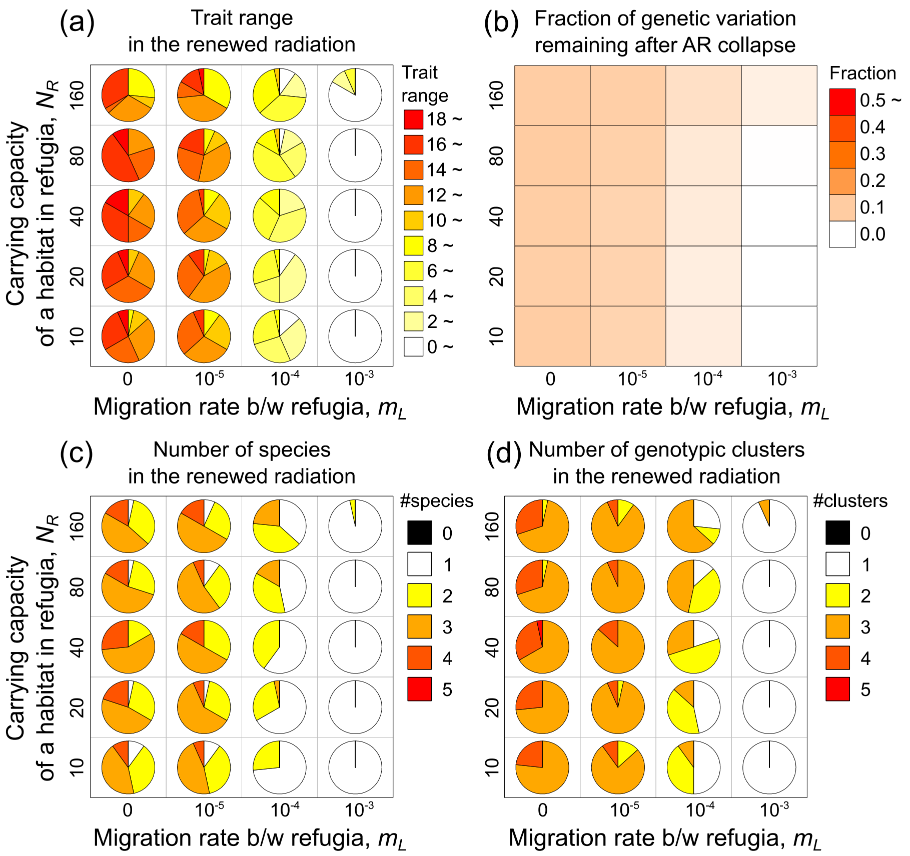

Figure S12. Effects of the strength of divergent selection in adaptive radiation φ_B and the population size in single habitats during the refugial phase N_R in the temporally repeated AR scenario. (a) Trait range at the generation 30000 across 30 simulation replications. (b) The fraction of remaining genetic variation (the number of SNPs) at the end of the refugial phase (the generation 20000) (averaged across 30 simulation replications). (c) Frequencies of the number of reproductively isolated and phenotypically distinct species at the generation 30000 across 30 simulation replications. (d) Frequencies of the number of genotypically distinct clusters of individuals at the generation 30000 across 30 simulation replications. Other parameters were set to default values (Table 1).

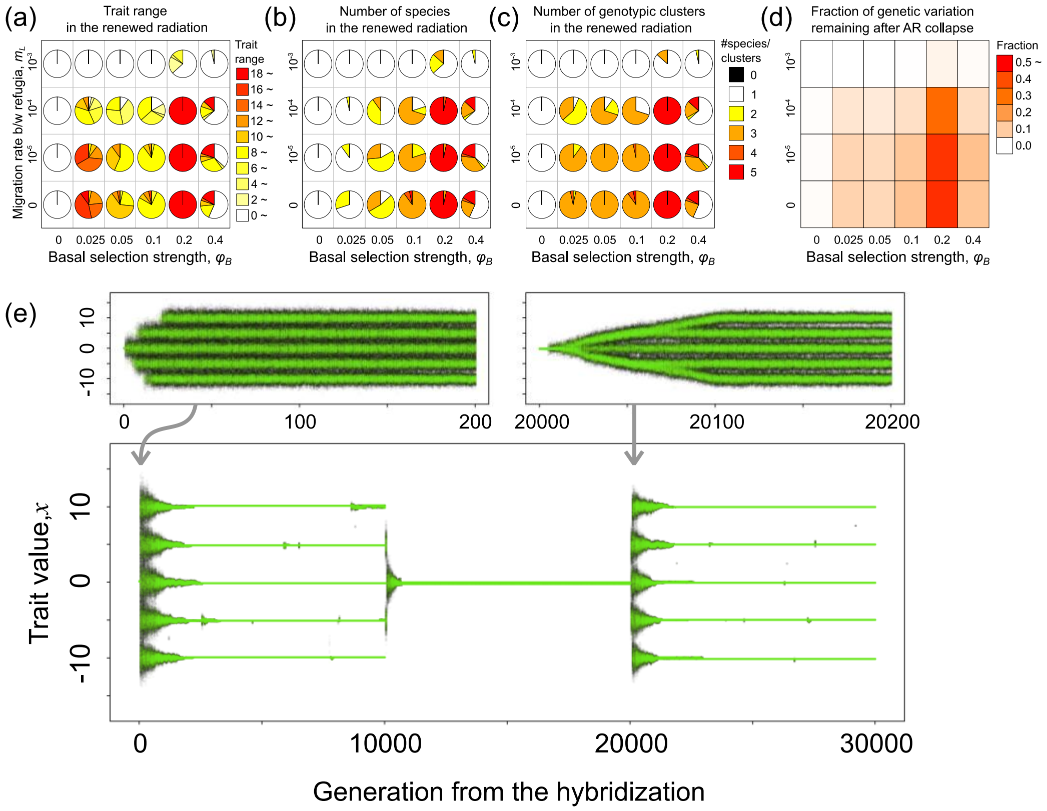

Figure S13. Simulation results with an alternative version of the temporally repeated AR scenario. In this scenario, we assumed that the environmental change led to a loss of divergent natural selection by causing shifts of optimal trait values in five habitats (x_{opt_H} for $H = H_{1, 2, \ldots 5}$) to the same value 0. To simulate such a dynamic change of optimal trait values, we assumed that the absolute value of optimal trait value in each habitat is not allowed to exceed a certain positive value d_{max} and that d_{max} gradually declines from 10 to 0 in the period of generations 10000 – 10100. When the value of d_{max} became smaller than the optimal value in a habitat H (x_{opt_H}), the value of x_{opt_H} was updated to d_{max}. Subsequently, environmental condition returns to the original state in the period of generations 20000 – 20100. In this period, the value of d_{max} gradually increased from 0 to 10. When the value of d_{max} exceeded the current value of x_{opt_H} and if the original value of x_{opt_H} was greater than or equal to the current value of d_{max}, the value of x_{opt_H} was updated to d_{max}. The strength of natural selection was not changed throughout the simulation (i.e. $\varphi_R = \varphi_B$). Therefore, refugial populations experienced a stabilizing natural selection with only a single optimal phenotype value 0. Simulations of this scenario with systematically varied parameter values revealed that the reestablishment of adaptive radiation could occur only when (i) migration rates between three patches during the refugial phase was low and (ii) divergent natural selection in the period before and after the refugial phase was moderately strong (panels a – c). These results are analogous to the results in our default version of simulation (Fig. 5). When natural selection in periods before and after the refugial phase was very weak (φ_B =

0.025), some extent of phenotypic diversity could have been reestablished after the collapse of radiation, but the reestablishment of species diversity was unlikely. This is because phenotypic diversification was easy under weak natural selection that produce only shallow fitness valleys between optimal phenotypes of five habitat, while immigrant inviability, the only mechanism for reproductive isolation of species in our model, could not be strong enough to separate ecologically distinct populations when natural selection was weak. Panel (e) shows an example of evolutionary dynamics in which adaptive radiation was rapidly reestablished after the environmental condition returned to the original state ($\varphi_B = 0.2$, $m_R = 0$). Other parameters were set to the default values (Table 1).

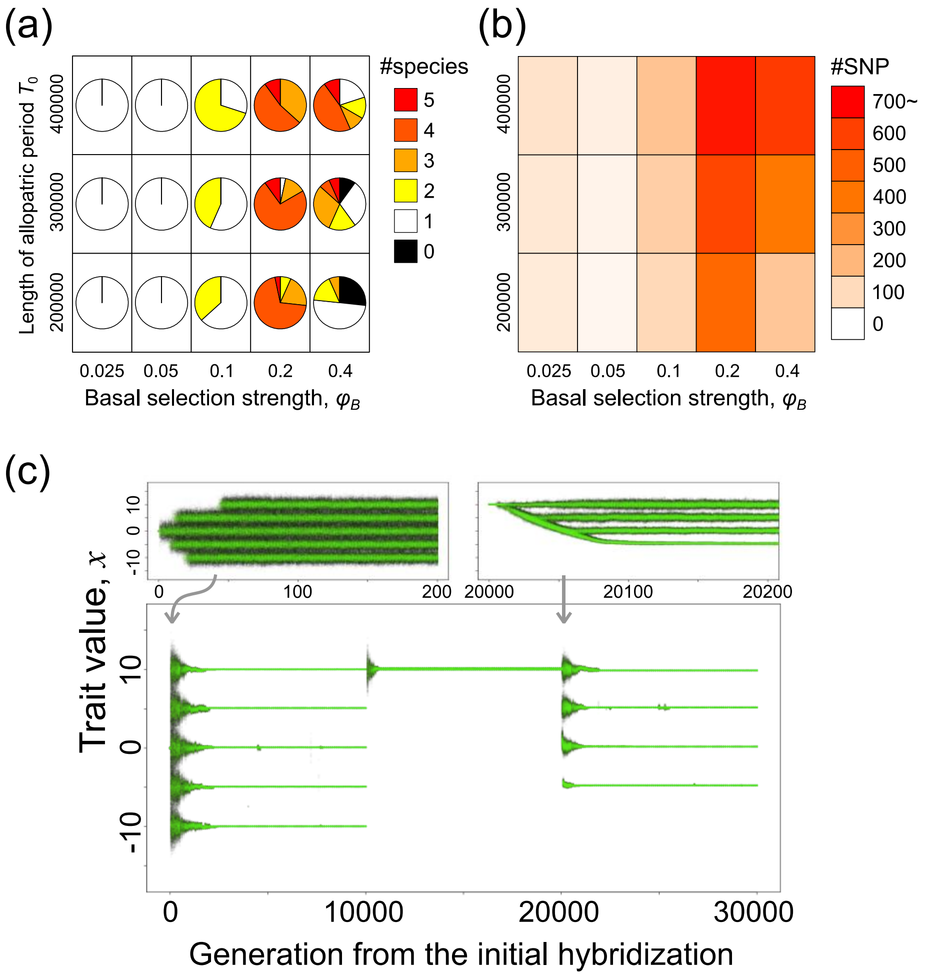

Figure S14. Simulation results with an alternative version of the temporally repeated AR scenario in which the environmental condition during the refugial period exerts natural selection favoring an extreme phenotype. The environmental change was modeled in the same way as the simulation of the Fig. S13 expect for that the optimal trait value of five habitats during the refugial phase was set to an extreme value ($x_{opt\_H} = 10$). (a, b) Results of simulations systematically varying the basal strength of divergent selection φ_B and the length of allopatric period before hybridization T_0. (a) The number of species at generation 30000 (N = 30 runs for each condition). (b) The extent of evolutionary potential at the end of the refugial phase. Each square shows the #SNPs at generation 20000. (c) An example of evolutionary dynamics. Parameters: $\varphi_B = 0.2$; other parameters: default values in Table S1. An extreme phenotype ($x \approx 10$) has fixed in all three refugia during the refugial phase. However, gradual environmental change during generations 10000 to 10100 caused speciation reversal (i.e. merge of formerly ecologically isolated incipient species into a hybrid swarm) in three refugia, which enabled

genetic differentiation among them through fixation of different genotypes associated with similar phenotypes. After the renewal of environmental variation, secondary admixture of three refugial sub-lineages reestablished phenotypic variation through transgressive segregation, thereby enabled reestablishment of incipient species specialized to each habitat type. However, reestablishment of specialists for habitats H_4 and H_5 was less likely compared to the case with the intermediate refugial environment (Fig. S13), due to the increased distance between the optimal phenotype in the refugial environment and in habitat H_4 and H_5.
